# Leucocyte- and platelet-rich fibrin as a rescue therapy for small-to-medium-sized complex wounds of the lower extremities

**DOI:** 10.1186/s41038-019-0149-0

**Published:** 2019-05-06

**Authors:** Kadri Ozer, Ozlem Colak

**Affiliations:** 1 Aydin State Hospital, Plastic, Reconstructive and Aesthetic Surgery Clinic, 09100 Aydin, Turkey; 2 Istanbul Okmeydani Training and Research Hospital, Plastic, Reconstructive and Aesthetic Surgery Clinic, 34384 Istanbul, Turkey

**Keywords:** Leukocyte- and platelet-rich fibrin, Complex wound, Bare bone, Bare tendon

## Abstract

**Background:**

Generally, advanced wound care resources are applied for complex wounds that pose a challenge to the medical and nursing teams. In this report, the use of leukocyte- and platelet-rich fibrin (L-PRF) is emphasized for complex wounds as an alternative, simple, inexpensive, time-saving process that does not require hospitalization and has a healing potential over that of bare soft tissue, including bone, tendon, and ligaments. The aim of this study is to extend the use of L-PRF in small-to-moderate-sized complex wounds of lower extremities in which L-PRF maintains the sensitive structures viable.

**Methods:**

Between January 2016 and December 2017, 17 small-to-moderate-sized complex wounds of lower extremities treated with L-PRF were recruited from the plastic and reconstructive surgery clinic in Aydin State Hospital, Turkey. The treatment was administered twice per week in the outpatient clinic. Depending on the size and extension of the complex wound, two to five blood samples were collected into 8.5 ml dry, glass vacuum tubes with no anticoagulant, and samples were immediately centrifuged at 1630×*g* for 5 min to obtain L-PRF. Complete healing was defined as the day of complete wound epithelialization.

**Results:**

The median values of the initial wound size and wound duration were 12 cm^2^ (interquartile range, 6 to 23 cm^2^) and 8 months before first admission (interquartile range, 4 to 18 months), respectively. All wounds showed significant improvements after L-PRF therapy and full closure after a median of 18 months, with an interquartile range of 11 to 34 months of L-PRF applications. There were recurrences of wounds during the first 6 months after therapy. No adverse events were observed.

**Conclusions:**

Our results add to the growing evidence that L-PRF treatments protect and maintain bare soft tissue structures viable, facilitate the formation of granulation tissue and epithelization, and remarkably reduce the need for additional soft tissue surgeries in small-to-medium-sized complex wounds.

**Electronic supplementary material:**

The online version of this article (10.1186/s41038-019-0149-0) contains supplementary material, which is available to authorized users.

## Background

Non-healing ulcers (unresponsive to initial therapy or persisting despite appropriate care and standard treatment) represent a substantial financial burden on the health care system [1]. “Advanced wound care methods” are considered when standard treatments have failed. In developed countries, it is estimated that 1 to 2% of the population will experience a chronic wound during their lifetime [2]. According to a new report, the global market for advanced wound care products will reach US$ 16.0 billion by 2022 [3]. The most common encountered chronic wounds are ulcers of the lower extremities, which usually last, on average, 12 to 13 months and therefore remain a major workload problem for clinicians [4]. Moreover, the biggest repercussions of such wounds are the decrease in quality of life and productivity of patients. In working patients, leg ulcerations are correlated with loss of time from work, loss of jobs, and adverse effects on finances [2].

Chronic wounds are classically defined as wounds that have failed to proceed through an orderly and timely reparative process to produce anatomic and functional integrity over a period of 3 months [5]. Although this definition has frequently been used, the term “chronic wound” only means that more time is needed to heal the wound. Hence, it could be argued that this term is not a good for characterizing the complexity of the problem [6]. Consequently, it would be better to use the term “complex wounds” rather than “chronic wounds” to describe well-known, difficult wounds that challenge medical and nursing teams regardless of whether they are acute or chronic [6].

Management of complex wounds has undergone major developments over the past decade, and the interest in the field of wound care has led to advancements of the use of tissue engineering and biological products. Recently, platelet-based products have gained significance and are currently some of the most commonly used biological products for wound healing. Although the use of platelet derivatives for the treatment of skin wounds has a five-decade history with various names [7], the first-known accepted description of the regenerative use of platelets was provided by Marx in 1998 as platelet-rich plasma (PRP) [8]. PRP was described as an autologous source of growth factors, such as platelet-derived growth factor, and the obtained growth factor-beta was transformed by sequestering and concentrating platelets via gradient density centrifugation [8]. After the working definition was provided by Marx et al. [8], platelet-based bioactive treatments gained in popularity in many areas, including dentistry, oral and maxillofacial surgery, dermatology, and cosmetic surgery. Described as an easily obtained, fast, effective, relatively cheap, and safe product, PRP has been the subject of increased clinical interest in the market [9]. Despite its widespread use, one of its reported drawbacks is the use of anticoagulation factors, which may cause a delay in normal wound-healing processes [10]. Additionally, ready-to-use commercially available disposable PRP preparations and separation kits could cost US$ 175–1150 per kit [11]. Therefore, the high costs and the need for specialized equipment to prepare PRP could critically reduce the use of autologous platelets in clinical practice [11]. Akhundov et al. commented that more simplified methods that do not require ad hoc and costly equipment would help to accumulate clinical data and introduce the method in a routine manner in clinical practice [11]. Therefore, it was necessary to develop manual methods for the preparation of low-cost PRP or to create different biological methods. Given these limitations, manual PRP techniques have evolved, and studies focused on developing a second-generation platelet concentrate. Consequently, a platelet concentrate lacking coagulation factors, which was later termed platelet-rich fibrin (PRF), was developed based on its anticipated properties in tissue regeneration and wound healing [10].

An autologous, solid, fibrin biomaterial was first introduced in 2000s. Leukocyte- and platelet-rich fibrin (L-PRF) has a very specific three-dimensional architecture (thick and dense polymerized fibrin strands), cell content, and distribution (97% of the platelets and > 50% of the leukocytes from the initial blood harvest) [12]. The growth factor content of L-PRF was logically expected to be much higher that of PRP as most platelets are activated in L-PRF clots [13]. An intact PRF membrane slowly releases 273.4 ± 15.3 ng transforming growth factor-ß1 (TGF-ß1), 6071 ± 773 pg vascular endothelial growth factor (VEGF) and 50.3 ± 6.3 ng platelet-derived growth factor-AB (PDGF-AB) over 7 days, which represent large amounts of these growth factors [13, 14].

In recent years, the interest in biological products, primarily in autologous platelet-rich preparations, has increased. The rationale of this study is related to the use of L-PRF concentrations, which is a relatively recent development that differs from other preparations given its potential for healing and neoangiogenesis [15]. In addition, the unique three-dimensional structure of L-PRF contains a known capacity of platelets, leukocytes, and growth factors that persist in the application site, providing superior prolonged action compared with other preparations [15]. In this report, the use of L-PRF on complex leg wounds is and presented as an alternative, simple, and low-cost method. The technique is fast and does not require hospitalization, leading to less time lost from work and good healing potential given that granulation tissue forms on bare bones, tendons, and ligaments in small-to-medium-sized wounds. The aim of this study is to extend the use of L-PRF in small-to-moderate-sized complex wounds, in which L-PRF maintains the sensitive structures viable and protects them from necrosis.

## Methods

### Study design and patient selection

A retrospective review was performed to evaluate the therapeutic effects of L-PRF on patients with complex wounds. Between January 2016 and December 2017, 17 small-to-moderate-sized complex wounds of lower extremities treated with L-PRF were recruited from plastic and reconstructive surgery clinic in Aydin State Hospital, Turkey. This institution is a station hospital where patients are sent from the surrounding counties, and the plastic surgery department is the only department that treats complex wounds. All the protocols used in this study were conducted according to the ethical guidelines of the 1975 Declaration of Helsinki and international regulations as reflected in the approval of the study by the Ethics Committee of Ankara Training and Research Hospital, Ankara, Turkey (0040/0408). Notably, informed consent was obtained from each patient. All cases reported in this study were treated in the outpatient clinic without the need for hospitalization or an operating room. Patients’ age and gender and the initial wound size, wound type, comorbidities, wound etiology, injured soft tissue structures, number of treatments, and duration of wound were identified and reviewed from their medical records.

The L-PRF treatment was applied twice per week until the wound was completely epithelialized. No extra specialized wound care was performed. Additionally, a topical antibiotic ointment (5 mg/g neomycin, 500 IU/g bacitracin) was occasionally applied as a prophylactic treatment when dressings were performed exclusively in the presence of an erythematous appearance around the wound. The primary endpoint was healing on consecutive days. Complete healing was defined as the day of complete wound epithelialization. Wound information was either collected by the medical personnel assessing the wounds or obtained from photographs of the wounds. In our clinical practice, wound measurements are made of the greatest length and width, and those measurements are multiplied to obtain the area of the initial wound size.

### Inclusion and exclusion criteria

The following inclusion criteria were used in this study: (a) patients over 18 years of age; (b) patients with well-known, difficult wounds that challenge medical and nursing teams regardless of whether they are acute or chronic; (c) patients with a chronic wound in need of another treatment that has not been healed by specialized wound care; (d) patients without any extra specialized wound care material other than L-PRF applications; and (e) patients with a minimum follow-up period of 6 months. The exclusion criteria were as follows: (a) patients with anemia or thrombocytopenia, (b) patients with suboptimal wound care before first admission, (c) patients with necrotic wounds because it was believed that platelets would be unable to penetrate the wound bed [16], and (d) patients with a disease and/or medication affecting platelet function and structure.

### L-PRF preparation and application

**Fig. 1:**
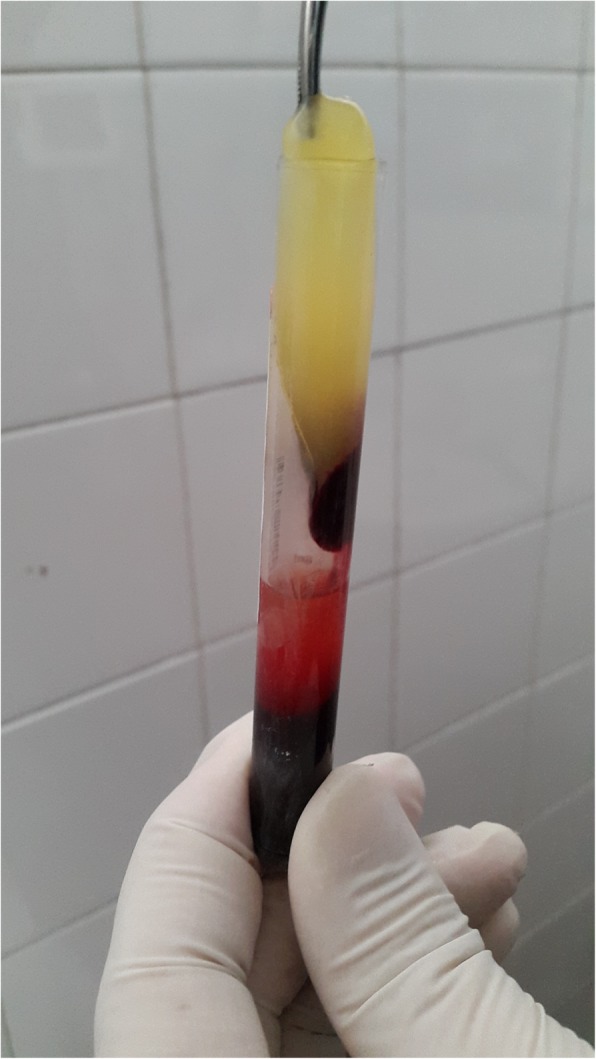
Leukocyte- and platelet-rich fibrin is observed in the middle layer and is stripped from the adjacent red blood layer with a sterile forceps before application


Two to five blood samples (depending on the size and extension of the defect to be filled) were collected in 8.5 ml dry, glass vacuum tubes with no anticoagulant and were immediately centrifuged at 1630×*g* for 5 min (see Additional file 1: Video S1). To prevent initiation of coagulation cascades before centrifugation and to allow natural transformation of the fibrin matrix during centrifugation, this step was performed as soon as blood was collected in the tubes. After centrifugation, three layers were observed. The basal layer consisted of red blood cells (most dense), the top layer consisted of non-cellular plasma (least dense), and the middle layer consisted of the L-PRF coagulate (medium density). Using sterile forceps, L-PRF was removed from the tube and stripped from the adjacent red blood cell layer (Fig. 1). With absorption of L-PRF serum into a gauze pad, a membrane rich in fibrin from the matrix that exhibited high resistance was obtained. During each visit, after mild irrigation and mild debridement of the wound, L-PRF treatment was applied and the wound was covered with a few thick pieces of gauze. All the procedures, including opening the wound, irrigation, debridement, preparation and application of L-PRF, and re-dressing, took approximately 10 to 15 min, on average.

**Additional file 1: Video S1.** Video demonstrating how to manually harvest and apply leukocyte- and platelet- rich fibrin. (MP4 200367 kb)


### Statistical analysis

Data were analyzed using SPSS 15.0 software (SPSS, Chicago, IL, USA). Data are presented as the median and interquartile ranges. Spearman’s rank correlation was used to determine the association between the initial wound size, duration of the wound, and number of L-PRF applications. Statistically significant was considered as *p* < 0.05.

## Results

### Demographic data and clinical characteristics of patients

L-PRF-treated wounds showed hastened healing with early wound contraction. Out of 17 patients, 11 patients (65%) were male and 6 (35%) were female, with a mean age of 59 years (ranging from 18 to 77 years) (Table 1). There were three acute wounds with a median duration of 2.5 months before first admission (interquartile range, 2 to 3 months) that occurred after trauma and 14 chronic wounds with a median duration of 8.5 months before first admission (interquartile range, 6 to 18.5 months) (Table 1). The median initial wound size at first admission was 12 cm^2^ (interquartile range, 6 to 23 cm^2^) (Table 1).

**Table 1:** Demographic data and clinical characteristics of the patients involved in the study.* PRF* platelet- rich fibrin

Patient	Gender	Age (years)	Comorbidities	Affected site	Initial wound size − length × width (cm^2^)	Duration of wound at first admission (months)	Number of PRF treatments	Wound healing time (weeks)
1	Male	72	Diabetes mellitus, hypertension, peripheral arterial disease	Left foot back	9 × 7	8	36	18
2	Male	76	Diabetes mellitus, hypertension, chronic venous insufficiency	Left distal lower leg lateral	5 × 7	18	48	24
3	Male	65	Diabetes mellitus, peripheral arterial disease	Right middle lower leg anterior	6 × 2	4	8	4
4	Male	18	Distal flap necrosis after posttraumatic flap surgeries	Right middle lower leg anterior	3 × 2	2.5	52	26
5	Male	43	Non-healing gunshot injury	Right middle lower leg posterior	5 × 3	2	18	9
6	Female	58	Diabetes mellitus, hypertension, peripheral arterial disease	Left distal lower leg medial	4 × 2	9	12	6
7	Female	63	Diabetes mellitus, hypertension, peripheral arterial disease	Right foot medial plantar	2 × 2	7	12	6
8	Male	56	Arteriovenous malformation, hypertension, uncontrolled psoriasis, chronic venous insufficiency	Right foot heel	5 × 5	240	60	30
9	Female	60	Diabetes mellitus, peripheral arterial disease	Right distal lower leg medial	2 × 1	8	8	4
10	Female	65	Diabetes mellitus, hypertension, arterial stenosis	Left distal foot lateral	6 × 3	6	20	10
11	Male	48	Chronic obstructive pulmonary disease, hypertension, necrosis after tumor resection	Left foot dorsomedial	3 × 2	4	12	6
12	Male	65	Diabetes mellitus, peripheral arterial disease	Left middle lower leg medial	4 × 3	6	10	5
13	Female	58	Peripheral arterial disease, cardiac valvular disease	Right foot dorsolateral	3 × 3	12	12	6
14	Male	64	Chronic obstructive pulmonary disease, hypertension	Right proximal leg anteromedial	5 × 5	20	32	16
15	Male	58	Peripheral arterial disease, chronic venous insufficiency	Right distal lower leg medial	5 × 3	18	24	12
16	Male	77	Diabetes mellitus, hypertension, chronic venous insufficiency, posttraumatic non-healing wound	Left calcaneal medial	2 × 2	3	8	4
17	Female	62	Diabetes mellitus, chronic venous insufficiency	Left metatarsophalangeal joint lateral	7 × 3	36	26	13

The median number of L-PRF applications was 18, with an interquartile range of 11 to 34 months (Table 1). The correlation between the initial wound size and the number of L-PRF applications was statistically significant (*r*_s_ = 0.699, *p* = 0.002). However, no correlation was found between the duration of the wound and the number of L-PRF applications (*r*_s_ = 0.445, *p* = 0.73). There was a statistically significant positive correlation between the initial wound size and the duration of the wound (*r*_s_ = 0.524, *p* = 0.031).

**Fig. 2:**
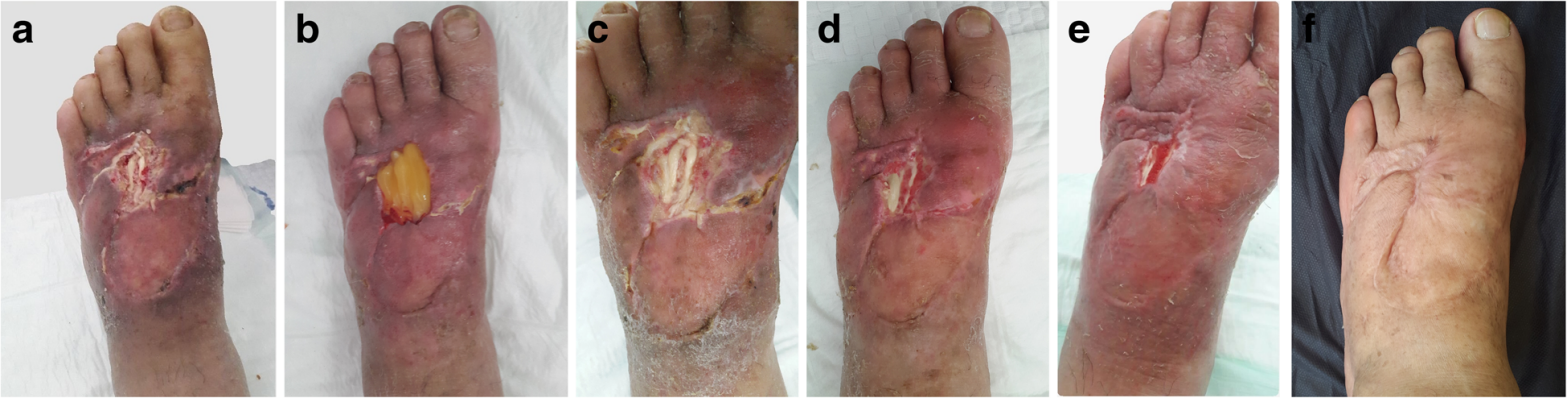
A 72-year-old man presented with necrosis of 80% of the total dorsal area of his foot after a soft tissue infection due to uncontrolled diabetes mellitus. **a** Bare tendons of the dorsal foot defect after sharp debridement due to distal flap necrosis, and **b** application of leukocyte- and platelet-rich fibrin (L-PRF) over the defect. **c** After five applications of L-PRF, a small granulation tissue started to appear with viable exposed tendons. **d** Six weeks after the first application, the wound contracted and the formed granulation tissue almost covered the whole bare tendon at the medial side. **e **Eight weeks after the first application, the wound contracted and greater than 50% of the initial wound was epithelized. **f **After 18 weeks of L-PRF application, a completely healed complex wound was obtained with no complications

**Fig. 3:**
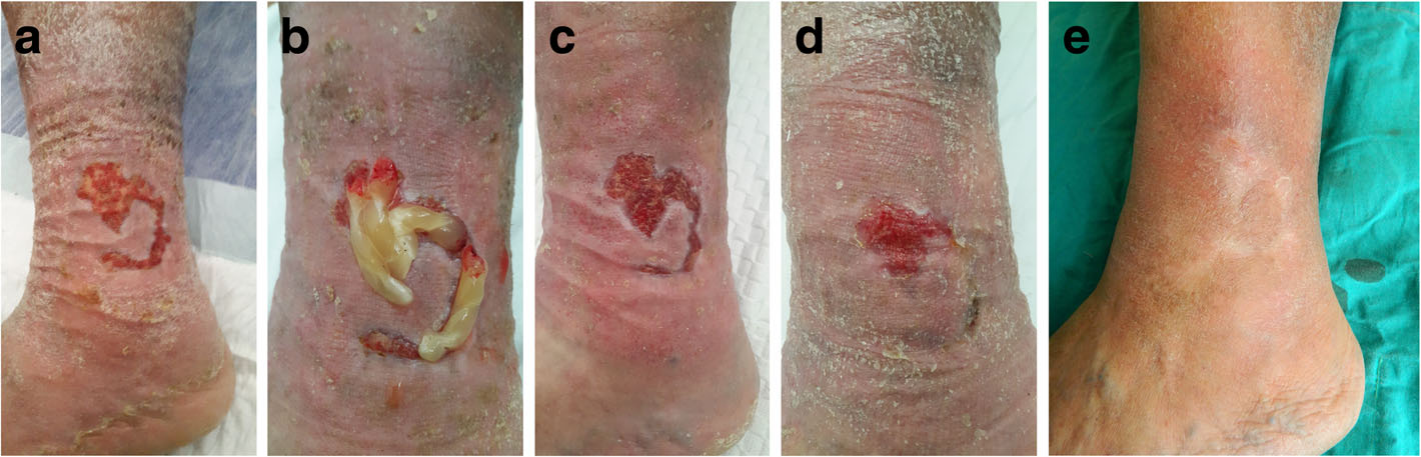
A 76-year-old-male presented with a one-and-a-half-year history of a chronic venous ulcer on his left lower leg. **a** The wound was initially managed with classical dressings prior to arrival at our clinic. **b** After six applications of leukocyte- and platelet-rich fibrin (L-PRF), **c** wound granulation closed over the wound. **d **With 24 applications of L-PRF, a good level of wound contraction was noted and the wound was nearly epithelized. **e** The complex chronic venous leg wound was uneventfully healed

**Fig. 4:**
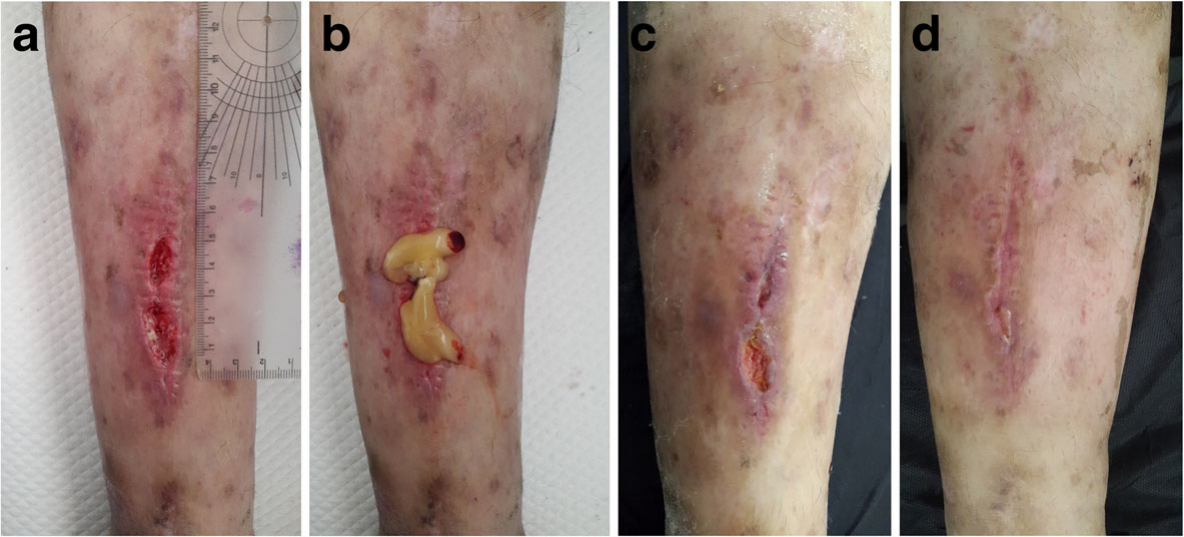
A 65-year-old-male presented with a 4-month history of a pretibial non-healed wound secondary to a trauma on his right lower leg. **a** Complex wound due to advanced peripheral arterial disease with diabetic dermopathy on the pretibia with an exposed bony part on the lower part after a sharp debridement. **b** After two applications of leukocyte- and platelet-rich fibrin (L-PRF), **c** wound granulation closed the bare bone. **d** After eight L-PRF applications, the wound was completely healed

**Fig. 5:**
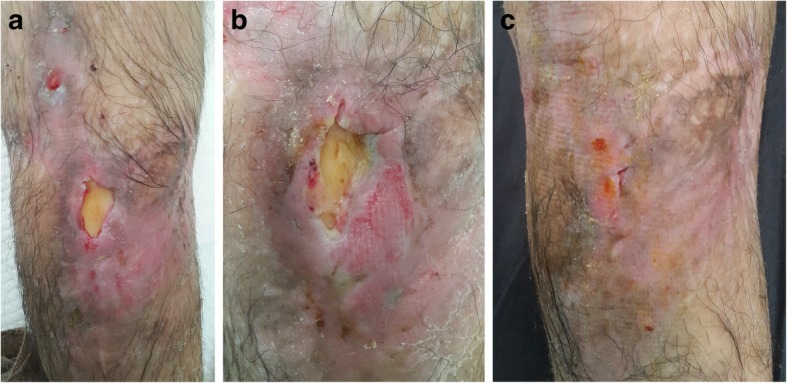
A healthy young male presented with a complex wound, including bare bone and peripherally unqualified skin and soft tissue, after tibial fracture surgeries to treat a motorbike injury. **a** After unsuccessful surgical operations, the patient did not prefer to undergo another surgery. **b** Leukocyte- and platelet-rich fibrin treatment was subsequently applied to the patient, and the wound gradually started to contract. **c** Approximately 6 months later, the defect was uneventfully closed

**Fig. 6:**
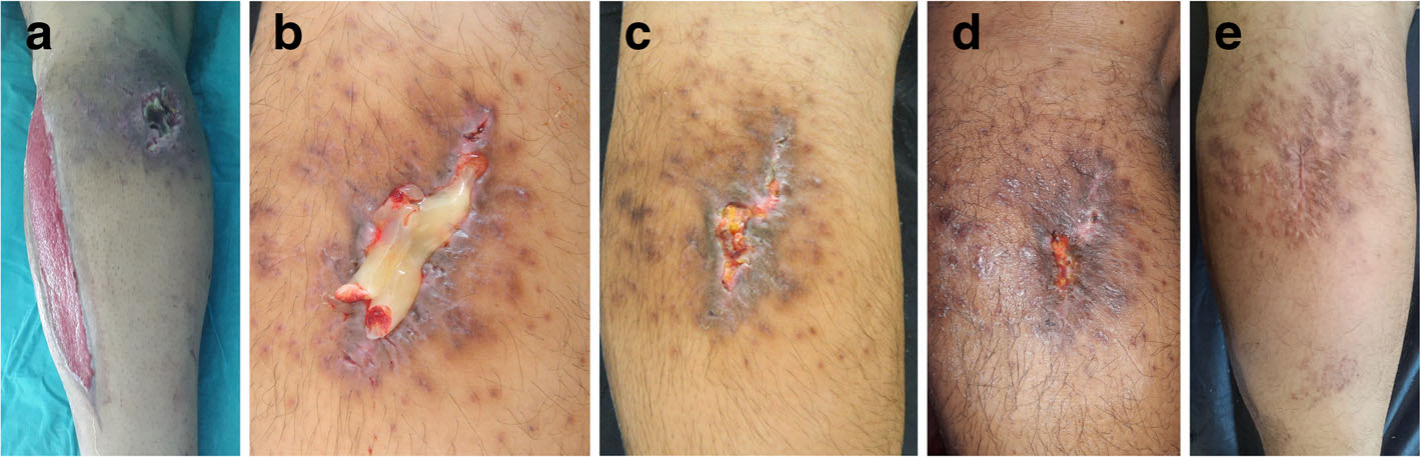
A 43-year-old-male presented with a non-healing gunshot injury. **a** Granulation tissue of the fasciotomy area after treatment with a vacuum-assisted closure system. In this healing period, the non-healed area was observed. **b** Leukocyte- and platele-rich fibrin (L-PRF) application on the complex wound area of the gunshot injury. **c** Wound healing after six applications of L-PRF, **d** and wound healing after 12 applications of L-PRF. **e** The complex wound was uneventfully healed after 18 applications of L-PRF

**Fig. 7:**
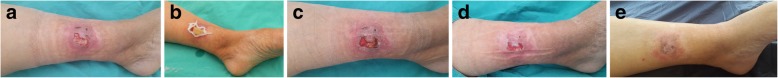
A 58-year-old-female presented with a peripherally erythematous left distal lower leg ulcer from an unknown cause. **a** The wound remained unhealed for 9 months. **b** Due to erythema, a topical ointment of neomycin and bacitracin was used to surround the leukocyto- and platelet-rich fibrin (L-PRF) application area. **c** Wound healing after two applications of L-PRF, **d** and wound healing after six applications of L-PRF. **e** The complex non-healed ulcer was successfully healed after 12 applications of L-PRF

Some of the cases are presented in Figs. 2, 3, 4, 5, 6, and 7. There was no wound recurrence for at least 6 months after therapy. Notably, adverse events related to therapy were not observed. A topical antibiotic ointment (5 mg/g neomycin sulfate, 500 IU/g bacitracin) was occasionally used in two patients. The ointment was applied around the L-PRF application when dressings were placed as a prophylactic treatment due to the erythematous appearance of the wound. However, no infection was observed during the treatment period. Final photographs of one patient were not found in the archives, and he did not answer our phone calls for a control visit and photographing (Case No: 8). No other complications and/or events were noted in the study.

### Case samples

Figure 2 shows a 72-year-old fit and independent man with a history of diabetes mellitus complicated by a soft tissue infection on his left foot extending to the lower leg. After hospitalization and intravenous antibiotic therapy in the infectious disease unit, 80% of the total dorsal area of the foot was affected by necrosis. The patient underwent surgical debridement and transposition flap surgeries with skin grafting of the donor sites. Due to distal flap necrosis, the bare tendons of the foot were exposed after sharp debridement (Fig. 2a). We performed L-PRF treatment twice per week (Fig. 2b). During each visit, the bare tendons were viable and white in color, unlike those in other commercial dressing materials (Fig. 2c–e). A completely healed foot was obtained without any observed complications (Fig. 2f). Similar results were observed in a 76-year-old-male with multiple comorbidities who presented with a one-and-a-half-year history of a chronic venous ulcer on his left lower leg (Fig. 3a–e). An interesting case of a non-healed wound secondary to trauma in a 65-year-old-male who had an advanced peripheral arterial disease with diabetic dermopathy was also observed, and the wound was successfully healed after 8 L-PRF applications (Fig. 4a–d). An example of an acute complex wound sample was a healthy young male who sustained a right tibial fracture from a motorbike injury, requiring surgical open reduction and internal fixation by a plate and screws. Additionally, he underwent consecutive flap surgeries, including a hemisoleus muscle flap with skin grafting and a transpositional fasciocutaneous flap due to recurrent distal necrosis of the flaps (Fig. 5a). After unsuccessful surgeries, the patient did not prefer to undergo another surgery. L-PRF treatment was subsequently applied to the patient. The wound was observed to contract after application (Fig. 5b). Approximately 6 months later, the defect was uneventfully closed (Fig. 5c).

## Discussion

Bioactive substances and their effects are hotly debated in the field of regenerative medicine. The main area of research seems to focus on the biology of platelets and leukocytes in wound-healing processes. Unfortunately, many studies mainly focus on growth factors. The fibrin architecture and the leukocyte content of these products are also often neglected [17]. The presence of leukocytes has a strong impact on the biology of these products not only given their immune and antibacterial properties but also their major key roles in the wound-healing process and local factor regulation [17].

The beneficial effect of L-PRF membranes in the healing of complex leg wounds can be explained by their high concentration of platelets and leukocytes together with the long-term release of growth factors specific to the L-PRF matrix [7]. The presence of a fibrin matrix enhances the delivery of growth factors over the wound area. Unlike PRP, L-PRF does not dissolve quickly; it dissolves over hours after application. Hence, L-PRF sustains a very significant slow release of key growth factors for days, which means that PRF stimulates its environment for a significant amount of time during the wound-healing process [14]. We hypothesize that the slow release of key growth factors during the first week may explain the positive results of PRF that we observed in our patients: “maintaining the viability of bare and vulnerable tissues such as tendon, bone, and/or ligaments.” In an *in vitro* study that included endothelial cell cultures and chick embryo chorioallantoic membrane assays, PRF preparations were somewhat more potent in angiogenesis than PRP preparations [18]. Leukocytes are among the main motivators of bone and soft tissue regeneration and contribute to the release of the angiogenic and lymphogenic factors responsible for cellular crosstalk in the tissue regeneration process [19]. Accordingly, without leukocytes, sophisticated cell–cell communication for tissue regeneration is not possible [19]. The increased presence of these cells potentially influences the differentiation of macrophages, which are key cells derived from the myeloid lineage and are implicated in growth factor secretion during wound healing, including TGF-beta, PDGF, and VEGF [10, 20, 21].

On the other hand, fibroblasts are the most influential cells in the production of collagen and other extracellular matrix components. Fibroblasts within chronic wounds have been shown to arrest the cell cycle [22]. For the initiation of wound-healing processes, it is important to induce the activation of the arrested cell cycles of fibroblasts within chronic wounds. Fibroblast proliferation is precisely regulated by cell cycle regulatory proteins, which are composed of two protein classes, cyclins and their kinase partners cyclin-dependent kinases (Cdks). Fibroblasts are strongly reactive to growth factors, including fibroblast growth factor-beta, epidermal growth factor, and PDGF. Notably, the expression of cyclins and cyclin-dependent kinase proteins increases in response to a high concentration of platelet-based suspensions [22]. These suspensions induced the upregulation of type I collagen as well as increased cell migration, proliferation rates, and expression of G1 cell cycle regulatory proteins, such as cyclin A, Cdk2, and cyclin E, in human skin fibroblasts [22].

The main controversy regarding L-PRF involves whether the presence of leukocytes has a beneficial or detrimental effect. Leukocytes produce catabolic cytokines that may impair tissue healing. On the other hand, some authors believe that leukocytes provide antimicrobial effects and natural protection against allergic responses [23, 24]. Although L-PRP and L-PRF are rich in leukocytes, increased total amounts of IL-1β were observed in blood clots with L-PRF and L-PRP due to the partial loss of leukocytes during L-PRP and L-PRF preparations [25]. The interleukin (IL)-1β concentration was previously found to be positively correlated with neutrophils and monocytes in L-PRP concentrates [26]. One study reported that more than half of leukocytes were trapped in PRF membranes; small lymphocytes were mainly collected but were not correlated with inflammation [12].

Leukocytes are also involved in the communication between precursor cells and mesenchymal cells with regard to bone formation [19]. The strongest induction of mesenchymal stem cell migration was observed in response to L-PRF, which may suggest the complexity of growth factors and cell interactions in cellular processes during tissue healing [25]. It should be noted that a pure growth factor or cytokine could have an inconsistent effect compared with the cocktail of factors present in wound healing. Ultimately, it can be hypothesized that L-PRF may provide a continuous and prolonged growth factor and cytokine system that involves a cascade of complex, orderly, and elaborate events with an acceptable environment for tissue injuries.

### Limitations

An acknowledged limitation of this study is the lack of a control group for L-PRF applications. A prospective study including patients undergoing L-PRF treatment compared with either another treatment or saline could be possible. However, it could be argued that comparing a treatment modality with a no treatment response control group would be unwise and unethical. Notably, L-PRF applications were performed in all of our patients with non-healed complex wounds despite their current wound care. On the other hand, while L-PRF may be considered helpful in wound healing and furthermore may essentially bypass some of the limitations of commercial single-growth factors, it is not the “holy grail” of wound healing. Other limitations of this study include the small sample size of the study and the single-center nature of the study.

## Conclusions

In our study, we used L-PRF to treat complex wounds in lower extremities. Tendon, and/or bone exposure commonly occurs in wounds of lower extremities. In addition, it could be challenging to form healthy granulation tissue by simple dressings in such exposed tissues while maintaining the viability of vulnerable tissues. For such wounds, an advanced therapy is recommended in the literature for those who do not respond to standard treatments within 4 weeks. Additionally, if a patient has any additional morbidities or problems, which can make surgery impossible and difficult, the clinician should seek alternative options other than surgery in small-to-medium-sized complex wounds. Due to the idealistic concept of *primum non nocere*, the clinician may experience a contradiction between doing no harm and doing better. Therefore, in these circumstances, L-PRF could represent a good alternative for small-to-medium-sized complex wounds because it can be prepared in a user-friendly manner with autologous, inexpensive, effective, and protective dressing materials and does not require any hospitalization. In conclusion, our results contribute to the growing evidence regarding the treatment modality of L-PRF. L-PRF protects and maintains the exposed tissues viable to facilitate the formation of granulation tissue, increase epithelization, and reduce the need for additional soft tissue surgery in small-to-medium-sized complex wounds.

## Abbreviations

Cdks: Cyclin-dependent kinases
L-PRF: Leukocyte- and platelet-rich fibrin
PDGF-AB: Platelet-derived growth factor-AB
PRP: Platelet-rich plasma
TGF-ß1: Transforming growth factor-ß1
VEGF: Vascular endothelial growth factor
